# Venom-gland transcriptomic, venomic, and antivenomic profiles of the spine-bellied sea snake (*Hydrophis curtus*) from the South China Sea

**DOI:** 10.1186/s12864-021-07824-7

**Published:** 2021-07-08

**Authors:** Hong-Yan Zhao, Lin Wen, Yu-Feng Miao, Yu Du, Yan Sun, Yin Yin, Chi-Xian Lin, Long-Hui Lin, Xiang Ji, Jian-Fang Gao

**Affiliations:** 1 Hangzhou Key Laboratory for Animal Adaptation and Evolution, College of Life and Environmental Sciences, Hangzhou Normal University, Hangzhou, 311121 Zhejiang China; 2 Hainan Key Laboratory of Herpetological Research, College of Fisheries and Life Science, Hainan Tropical Ocean University, Sanya, 572022 Hainan China; 3 MOE Key Laboratory of Utilization and Conservation for Tropical Marine Bioresources, Hainan Tropical Ocean University, Sanya, 572022 Hainan China; 4 Jiangsu Key Laboratory for Biodiversity and Biotechnology, College of Life Sciences, Nanjing Normal University, Nanjing, 210023 Jiangsu China; 5 College of Life and Environmental Sciences, Wenzhou University, Wenzhou, 325035 Zhejiang China

**Keywords:** Omics, *Hydrophis curtus*, Snake venom, Transcriptome, Proteome, Antivenomic, Positive selection

## Abstract

**Background:**

A comprehensive evaluation of the -omic profiles of venom is important for understanding the potential function and evolution of snake venom. Here, we conducted an integrated multi-omics-analysis to unveil the venom-transcriptomic and venomic profiles in a same group of spine-bellied sea snakes (*Hydrophis curtus*) from the South China Sea, where the snake is a widespread species and might generate regionally-specific venom potentially harmful to human activities. The capacity of two heterologous antivenoms to immunocapture the *H. curtus* venom was determined for an in-depth evaluation of their rationality in treatment of *H. curtus* envenomation. In addition, a phylogenetic analysis by maximum likelihood was used to detect the adaptive molecular evolution of full-length toxin-coding unigenes.

**Results:**

A total of 90,909,384 pairs of clean reads were generated via Illumina sequencing from a pooled cDNA library of six specimens, and yielding 148,121 unigenes through de novo assembly. Sequence similarity searching harvested 63,845 valid annotations, including 63,789 non-toxin-coding and 56 toxin-coding unigenes belonging to 22 protein families. Three protein families, three-finger toxins (3-FTx), phospholipase A_2_ (PLA_2_), and cysteine-rich secretory protein, were detected in the venom proteome. 3-FTx (27.15% in the transcriptome/41.94% in the proteome) and PLA_2_ (59.71%/49.36%) were identified as the most abundant families in the venom-gland transcriptome and venom proteome. In addition, 24 unigenes from 11 protein families were shown to have experienced positive selection in their evolutionary history, whereas four were relatively conserved throughout evolution. Commercial *Naja atra* antivenom exhibited a stronger capacity than *Bungarus multicinctus* antivenom to immunocapture *H. curtus* venom components, especially short neurotoxins, with the capacity of both antivenoms to immunocapture short neurotoxins being weaker than that for PLA_2_s.

**Conclusions:**

Our study clarified the venom-gland transcriptomic and venomic profiles along with the within-group divergence of a *H. curtus* population from the South China Sea. Adaptive evolution of most venom components driven by natural selection appeared to occur rapidly during evolutionary history. Notably, the utility of commercial *N. atra* and *B. multicinctus* antivenoms against *H. curtus* toxins was not comprehensive; thus, the development of species-specific antivenom is urgently needed.

**Supplementary Information:**

The online version contains supplementary material available at 10.1186/s12864-021-07824-7.

## Background

Sea snakes, the largest group of current marine reptiles, are widely distributed in many tropical and subtropical waters throughout the Indo-Pacific Ocean [1, 2]. Sea snakes display a diverse array of morphological, physiological, and behavioural traits modified for secondary adaptation to the marine environment; e.g., the venom has evolved to be biochemically simple despite comprising a pharmacologically toxic arsenal [3, 4]. It is generally believed that the adaptive evolution of snake venom composition and function is heavily propelled by natural selection from dietary shift, which might be accompanied by gene birth-and-death processes at the molecular level. A typical case is the shift of the marbled sea snake (*Aipysurus eydouxii*) to trophic specialization for fish eggs, which involved the dinucleotide deletion of neurotoxin genes and consequently the loss of primary neurotoxic activity in the venom [5]. Defining the venom profiles at the transcriptomic and proteomic levels is helpful for understanding the adaptive evolution and functional complexity of sea snake venom, as well as managing the envenomation caused by sea snakes and, exploring the medically important components and designing the antivenoms against sea snake venom. However, owing to the relatively ambiguous taxonomy of sea snakes, little attention has been paid to snakebites caused by sea snakes and few investigations have been conducted to uncover the profiles of sea snake venom at the ‘-omics’ level. Sea snake venom was previously shown to be mainly composed of three-finger toxins (3-FTx; 26.3–86.9%) and phospholipase A2s (PLA_2_s; 10.9–66.7%) that vary between and within species [3, 4, 6–10]. Generally, the diversity of the venom proteome is closely related to the profiles of toxin-coding genes, with the sequence conservation of these genes varying during evolutionary history [11]. Therefore, detecting the strength of natural selection on toxin-coding genes together with their homologs would be helpful in explaining the diversity of the venom proteome. Moreover, transcriptional and translational regulation along with post-translational modification is also considered to affect the diverse evolution of venom components. In particular, the abundance and type of toxin transcripts from the venom gland are highly concordant with the venom proteins in several snakes but extremely divergent in others [11–18]. For example, the toxin-coding genes detected in the venom gland of the spine-bellied sea snake (*Hydrophis curtus*; formerly *Lapemis curtus*) are not well correlated with the secreted venom proteins [6, 8, 19]. Nevertheless, such a divergence is deduced according to the venom-gland transcriptomic and venom proteomic data on *H. curtus* specimens from different regions, and still requires in-depth verification using specimens from the same region.

As most sea snakes are usually benign unless they are provoked, the incidence of sea snake bites is much lower than that caused by terrestrial venomous snakes [4, 20, 21]. Actually, over a half of the sea snake bites are related to professional fishing because sea snakes constitute a consistent by-catch of tropical trawl fisheries and the fishermen can get bitted by sea snakes when handling the fishing net [22–24]. It is estimated that significant envenomation and death occur in approximately 20 and 3% of sea snake bites, respectively [24], with the death rate reaching 50% if the victims are not properly treated [25]. Generally, the toxin constituents of sea snake venom are predominated by PLA_2_, 3-FTx and CRISP families, and express various biological/toxic activities, such as myotoxicity (PLA_2_) [26], postsynaptic neurotoxicity [27], and inhibit smooth muscle contraction and cyclic nucleotide-gated ion channels (CRISP) [28]. Nevertheless, the clinical manifestations of sea snake envenomation are mainly attributed to the 3-FTxs and PLA_2_s, which can induce systematic symptoms (e.g. paralysis, dysphagia, respiratory failure, and myonecrosis) [24, 25]. Only a single sea snake antivenom is currently commercially available, which is prepared against *Hydrophis schistosa* (formerly *Enhydrina schistosa*) venom by Seqirus (a division of Australian company CSL Limited) and is effective in the treatment of envenomation caused by a wide variety of sea snakes [29, 30]. In practice, however, not all victims envenomed by sea snakes receive an injection of sea snake antivenom. In China, for example, sea snakebites are predominantly caused by *H. curtus* and *Hydrophis cyanocinctus*, but the patients are either treated with traditional Chinese and western medicine or receive injections of many-banded krait *Bungarus multicinctus* and Chinese cobra *Naja atra* antivenoms [31, 32]. Nevertheless, the efficacy and safety of such therapeutic schedules based on antivenoms from phylogenetically related species have yet to be estimated thoroughly.

*Hydrophis curtus* is the most important of the 16 species of sea snakes in China in terms of the quantity and distribution [33]. This species potentially causes severe illness or death even though it is not defined as a medically important venomous snake [26]. Actually, WHO and the toxicologists are always vigorously appealing people and governments around the world to pay broad attention on snakebites, a neglected tropical disease, including those caused by *H. curtus*. Therefore, clarifying the global profiles of *H. curtus* venom would be helpful to understand the clinical symptoms of snakebites caused by *H. curtus*. Moreover, it is also necessary to qualitatively and quantitatively evaluate the availability of heterologous antivenoms in treatment of snakebites caused by *H. curtus*. In this study, we therefore applied a strategy combining next-generation sequencing technology and proteomic analysis to unveil the toxin profiles in the venom-gland transcriptome and venom proteome of *H. curtus* from the South China Sea. The strength of natural selection experienced during the evolutionary history of toxins was tested based on the identified toxin-coding unigenes. Moreover, antivenomic analysis, ELISA and western blotting were used to evaluate and compare the capacity of commercial *B. multicinctus* and *N. atra* antivenoms to immunocapture *H. curtus* venom components.

## Results

### Sequencing and de novo assembly

**Fig. 1:**
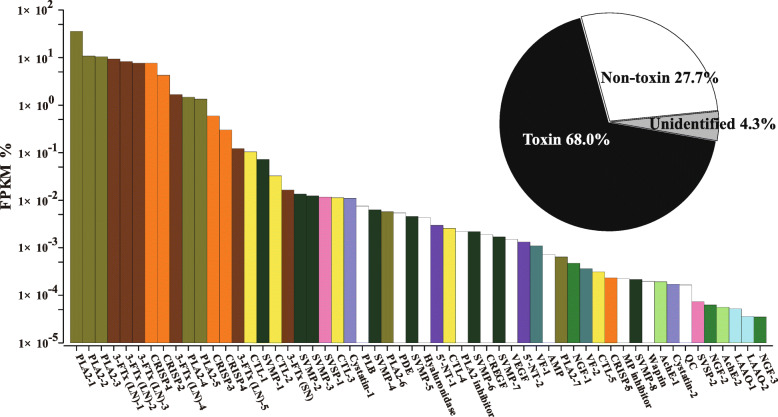
Venom-gland transcriptomic profiles of *H. curtus*. The details are listed in Additional Tables S1 and S2. FPKM, expected number of fragments per kilobase of transcript sequence per millions base pairs sequenced. The percentage of annotated (toxin and non-toxin) and unannotated (unidentified) unigenes in the whole transcriptome was listed in the inserted pie graph. 3-FTx, three finger toxin; PLA_2_, phospholipase A_2_; CRISP, cysteine-rich secretory protein; CTL, C-type lectin; SVMP, snake venom metalloproteinase; SVSP, snake venom serine proteinase; PLB, phospholipase B; PDE, phosphodiesterase; HA, hyaluronidase; 5′NT, 5′ nucleotidase; CREGF, cysteine-rich EGF-like domain; VEGF, vascular endothelial growth factor; VF, venom factor; AP, aminopeptidase; NGF, nerve growth factor; AchE, acetylcholinesterase; QC, glutaminyl-peptide cyclotransferases; LAAO, l-amino acid oxidase

A total of 90,909,384 pairs of clean reads were filtered from 93,226,644 pairs of raw reads generated from Illumina sequencing of the venom gland cDNA library of *H. curtus*, derived from six *H. curtus* individuals captured as by-catch from the South China Sea, and assembled into 284,495 transcripts (N50/N90 = 1763/290) using Trinity. Subsequently, 148,121 unigenes (N50/N90 = 2229/580) were clustered from these transcripts using Corset, among which 88,806 unigenes passed the quality filter (FPKM > 1). Following similarity alignment between unigenes and sequences in NCBI non-redundant protein (NR)/nucleotide (NT) and Uniprot databases (strict to Serpentes) using Diamond and BLAST, the assemblies were finally categorized into toxin (56), non-toxin (63,789), and unidentified (24,961) groups. The toxin group was expressed at markedly higher redundancies (50,456.48 FPKM/unigene) than those of the non-toxin group (18.02 FPKM/unigene). Moreover, the toxin group comprised only 56 unigenes but accounted for 68% of the total abundance expressed in FPKM of the *H. curtus* transcriptome, whereas the non-toxin and unidentified groups accounted for 27.7 and 4.3%, respectively (Fig. 1).

### Venom-gland transcriptomic profile

A total of 22 protein families were derived from 56 toxin-coding unigenes with partial and complete CDS using bioinformatics analyses (Fig. 1 and Additional Table S1). Three toxin families including PLA_2_ (59.71%), 3-FTx (27.15%), and cysteine-rich secretory protein (CRISP, 12.82%) appeared to dominate the toxin constituents. The remaining 19 toxin families were only expressed at low abundance with a total FPKM of 0.32%, including snake venom metalloproteinase (SVMP), C-type lectin (CTL), snake venom serine proteinase (SVSP), cysteine-type inhibitor (cystatin), phospholipase B (PLB), phosphodiesterase (PDE), hyaluronidase (HA), 5′ nucleotidase (5′NT), PLA_2_ inhibitor, cysteine-rich with EGF-like domain (CREGF), vascular endothelial growth factor (VEGF), venom factor, aminopeptidase, nerve growth factor (NGF), acetylcholinesterase (AchE), metalloproteinase inhibitor (MP), waprin, glutaminyl-peptide cyclotransferases (QC), and l-amino acid oxidase (Fig. 1). The relative abundance and sequences of toxin unigenes, arranged according to toxin family, are listed in Additional Tables S1 and S2.

### Venom proteomic profile

**Fig. 2:**
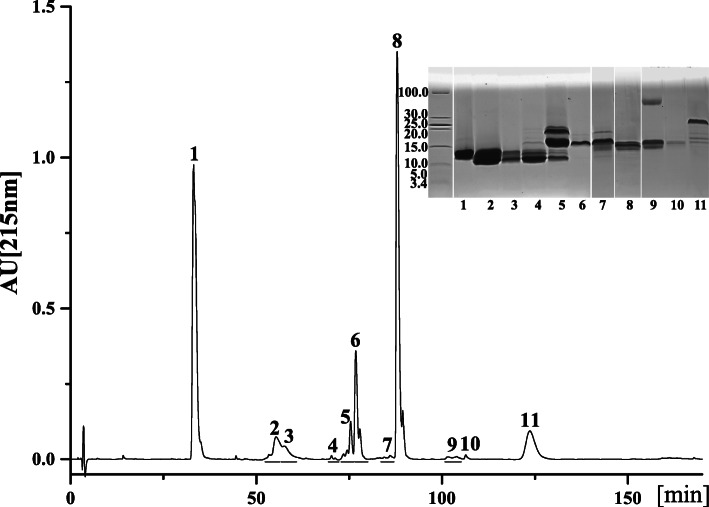
Characterization of the venom proteins of *H. curtus* from South China Sea. Three milligrams of total venom were applied to a C18 column, and separated as described on Materials and methods. Fractions were collected manually and submitted to molecular determination by SDS-PAGE under reduced conditions (original images were listed in Figure S1). Protein bands were excised, tryptic digested and analyzed by MALDI-TOF/TOF or nESI-MS/MS for their assignment to known protein families. The results are shown in Table 1

**Fig. 3:**
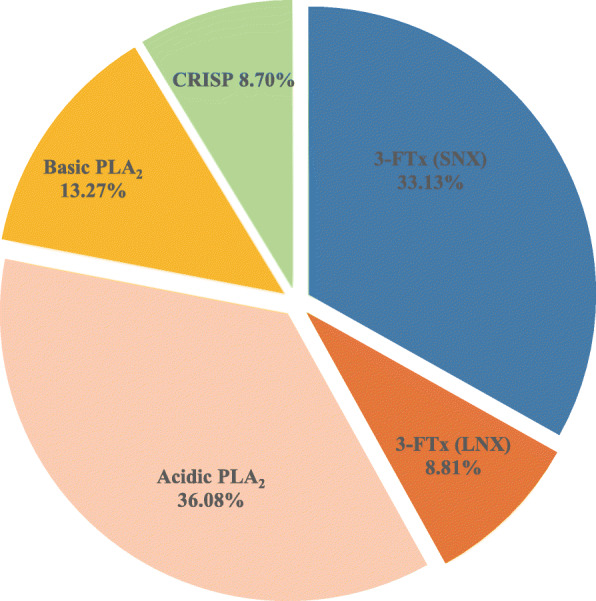
Venom proteomic profiles of *H. curtus*. The details are listed in Table 1. 3-FTx, three finger toxin; PLA_2_, phospholipase A_2_; CRISP, cysteine-rich secretory protein; LNX, long chain α-neurotoxin; SNX, short chain α-neurotoxin

Eleven chromatographic fractions (peaks) were resolved in crude *H. curtus* venom by RP-HPLC and 32 protein bands from gels were identified by MS analysis (Fig. 2 and Table 1). The proteomic analysis showed that *H. curtus* venom was dominated by three toxin families including PLA_2_ (49.36%), 3-FTx (41.94%), and CRISP (8.70%) (Fig. 3 and Table 1). Specifically, chromatographic peaks 1–4 contained 3-FTx accounting for 40.37% of the total venom protein, peaks 5–10 contained PLA_2_ (49.36%) at high abundance and 3-FTx (1.57%) at very low abundance, and peak 11 contained only CRISP. Additionally, acidic PLA_2_ (36.08%) and short-neurotoxin (SNX, 33.13%) were the predominant components in PLA_2_ and 3-FTx families, respectively.

**Table 1:** Assignment of the chromatographic fractions and electrophoretic bands from *H. curtus* venom to protein families

Peak	%	MW (kDa)	Peptide Ion		MS/MS-derived sequence	Protein family/species/accession/transcript ID
			m/***z***	***z***		
1	33.13	12.9	401.2	2	TWSDHR	3-FTx (SNX); *Hydrophis schistosus*; P68415; Hcu|50480
			650.8	2	GCGCPBVBPGXB	
			669.3	2	TCCNBBSSBPB	
			711.3	2	TTTNCAESSCYB	
			734.8	2	MTCCNBBSSBPB	
			742.8	2	MTCCNBBSSBPB	
			517.2	3	TTTNCAESSCYBB	
			775.3	2	TTTNCAESSCYBB	
2	3.05	13.3	1275.5	1	SWCDAFCSSR	3-FTx (LNX); *Hydrophis hardwickii*; A3FM53; Hcu|53057
			2190.0	1	THPYBPETCPPGBNXCYB	
	1.56	11.5	1275.5	1	SWCDAFCSSR	3-FTx (LNX); *H. hardwickii*; A3FM53; Hcu|54185
			1403.6	1	BSWCDAFCSSR	
			2055.9	1	DXNCCATDNCNTVANWB	
			2190.0	1	THPYBPETCPPGBNXCYB	
3	1.39	13.8	1245.5	1	SWCDAFCGSR	3-FTx (LNX); *H. hardwickii*; Q8UW28; Hcu|53057
	0.90	12.0	1245.5	1	SWCDAFCGSR	3-FTx (LNX); *H. hardwickii*; Q8UW28; Hcu|53057
4	0.14	16.8	1245.5	1	SWCDAFCGSR	3-FTx (LNX); *H. hardwickii*; Q8UW28; Hcu|53057
	0.03	14.7	1245.5	1	SWCDAFCGSR	3-FTx (LNX); *H. hardwickii*; Q8UW28; Hcu|53057
	0.04	13.8	1275.5	1	SWCDAFCSSR	3-FTx (LNX); *H. hardwickii*; Q8UW29; Hcu|51842
			1403.6	1	BSWCDAFCSSR	
			2053.0	1	TPYBPETCPPGBNXCYB	
	0.13	12.3	1275.5	1	SWCDAFCSSR	3-FTx (LNX); *H. hardwickii*; A3FM53; Hcu|54185
			1403.6	1	BSWCDAFCSSR	
			2055.9	1	DXNCCATDNCNTVANWB	
			2190.0	1	THPYBPETCPPGBNXCYB	
5	1.05	25.3	1336.6	1	XHDDCYGEAEB	PLA_2_; *H. schistosus*; P00610; Hcu|52733
			1514.7	1	NAYNNANYNXDTB	
			1878.9	1	NXVBFSYVXTCANHNR	
			1936.0	1	NXVBFSYVXTCANHNR	
			2785.2	1	SSXDYADYGCYCGAGGSGTPVDEXDR	
	0.33	21.1	1336.6	1	XHDDCYGEAEB	PLA_2_; *H. schistosus*; P00610; Hcu|52733
			1514.7	1	NAYNNANYNXDTB	
			1878.9	1	NXVBFSYVXTCANHNR	
			1936.0	1	NXVBFSYVXTCANHNR	
			2069.9	1	XHDDCYGEAEBBGCYPB	
			2785.2	1	SSXDYADYGCYCGAGGSGTPVDEXDR	
	1.14	16.9	1336.6	1	XHDDCYGEAEB	PLA_2_; *H. schistosus*; P00610; Hcu|52733
			1514.7	1	NAYNNANYNXDTB	
			1878.9	1	NXVBFSYVXTCANHNR	
			2785.1	1	SSXDYADYGCYCGAGGSGTPVDEXDR	
	0.15	14.8	1336.6	1	XHDDCYGEAEB	PLA_2_; *H. hardwickii*; Q8UW30; Hcu|52733
			1514.7	1	NAYNNANYNXDTB	
			1955.8	1	MXXYDYDCGSNGPYCB	
			2785.2	1	SSXDYADYGCYCGAGGSGTPVDEXDR	
	0.27	13.9	1275.5	1	SWCDAFCSSR	3-FTx (LNX); *H. hardwickii*; Q8UW29; Hcu|51842
			1403.6	1	BSWCDAFCSSR	
			2053.0	1	TPYBPETCPPGBNXCYB	
	1.16	12.6	1275.5	1	SWCDAFCSSR	3-FTx (LNX); *H. hardwickii*; Q8UW29; Hcu|51395
6	1.56	19.1	1514.7	1	NAYNNANYNXDTB	PLA_2_; *H. schistosus*; P00610; Hcu|52733
			1879.0	1	NXVBFSYVXTCANHNR	
			1936.0	1	NXVBFSYVXTCANHNR	
	8.34	16.9	1336.6	1	XHDDCYGEAEB	PLA_2_; *H. schistosus*; P00610; Hcu|52499
			1514.7	1	NAYNNANYNXDTB	
			1878.9	1	NXVBFSYVXTCANHNR	
			1936.0	1	NXVBFSYVXTCANHNR	
			2785.1	1	SSXDYADYGCYCGAGGSGTPVDEXDR	
7	0.33	18.9	1336.6	1	XHDDCYGEAEB	PLA_2_; *H. schistosus*; P00610; Hcu|52733
			1514.7	1	NAYNNANYNXDTB	
			1936.0	1	NXVBFSYVXTCANHNR	
	0.25	16.4	1336.6	1	XHDDCYGEAEB	PLA_2_; *H. schistosus*; P00610; Hcu|52733
			1514.7	1	NAYNNANYNXDTB	
			1879.0	1	NXVBFSYVXTCANHNR	
			1936.0	1	NXVBFSYVXTCANHNR	
			2785.2	1	SSXDYADYGCYCGAGGSGTPVDEXDR	
	0.12	14.5	1936.0	1	NXVBFSYVXTCANHNR	PLA_2_; *H. schistosus*; P00610; Hcu|52837
	0.15	12.0	1245.5	1	SWCDAFCGSR	3-FTx (LNX); *H. hardwickii*; Q8UW28; Hcu|53057
8	19.26	16.1	528.2	2	AFXCNCDR	PLA_2_; *H. hardwickii*; Q8UW31; Hcu|52837
			429.9	3	NMXBCANHGSR	
			644.3	2	NMXBCANHGSR	
			435.2	3	NMXBCANHGSR	
			652.3	2	NMXBCANHGSR	
			668.8	2	XHDDCYGEAEB	
			692.3	2	TAAXCFAGAPYNB	
			644.3	3	DNNDECBAFXCNCDR	
			965.9	2	DNNDECBAFXCNCDR	
			829.7	3	TAAXCFAGAPYNBENYNXDXNB	
			986.4	3	MTXDYMDYGCYCGTGGSGTPVDEXDR	
	5.03	15.4	1055.5	1	AFXCNCDR	PLA_2_; *H. hardwickii*; Q8UW31; Hcu|52837
			1383.7	1	TAAXCFAGAPYNB	
	10.67	14.4	1383.7	1	TAAXCFAGAPYNB	PLA_2_; *H. hardwickii*; Q8UW31; Hcu|52515
9	0.31	73.0	561.8	2	ENYNXDXNB	PLA_2_; *H. hardwickii*; Q8UW31; Hcu|52733
			587.3	2	MXBCANHGSR	
			516.6	3	ENYNXDXNBHCB	
			429.9	3	NMXBCANHGSR	
			652.3	2	NMXBCANHGSR	
			692.3	2	TAAXCFAGAPYNB	
			809.4	2	XHDDCYGEAEBXPA	
			680.8	2	GTGGSGTPVDEXDR	
			760.8	2	CGTGGSGTPVDEXDR	
			694.3	3	NXYBFBNMXBCANHGSR	
			699.7	3	NXYBFBNMXBCANHGSR	
			829.7	3	TAAXCFAGAPYNBENYNXDXNB	
			978.4	3	MTXDYMDYGCYCGTGGSGTPVDEXDR	
			983.7	3	MTXDYMDYGCYCGTGGSGTPVDEXDR	
			983.7	3	MTXDYMDYGCYCGTGGSGTPVDEXDR	
			1483.1	2	MTXDYMDYGCYCGTGGSGTPVDEXDR	
			1092.0	4	XPACNYMXSGPYYNXYTYDCVEHBXTCBDNNDECB	
			962.2	5	XHDDCYGEAEBXPACNYMXSGPYYNXYTYDCVEHBXTCB	
	0.29	15.9	1055.5	1	AFXCNCDR	PLA_2_; *H. hardwickii*; Q8UW31; Hcu|52733
			1383.7	1	TAAXCFAGAPYNB	
	0.19	14.6	528.2	2	AFXCNCDR	PLA_2_; *H. hardwickii*; Q8UW31; Hcu|52837
			561.8	2	ENYNXDXNB	
			644.3	2	NMXBCANHGSR	
			429.9	3	NMXBCANHGSR	
			652.3	2	NMXBCANHGSR	
			435.2	3	NMXBCANHGSR	
			668.8	2	XHDDCYGEAEB	
			692.3	2	TAAXCFAGAPYNB	
			774.3	2	ENYNXDXNBHCB	
			595.6	3	CCBXHDDCYGEAEB	
			965.9	2	DNNDECBAFXCNCDR	
			829.7	3	TAAXCFAGAPYNBENYNXDXNB	
			978.4	3	MTXDYMDYGCYCGTGGSGTPVDEXDR	
			1467.1	2	MTXDYMDYGCYCGTGGSGTPVDEXDR	
			989.1	3	MTXDYMDYGCYCGTGGSGTPVDEXDR	
			1475.1	2	MTXDYMDYGCYCGTGGSGTPVDEXDR	
			1158.5	3	XPACNYMXSGPYYNXYTYDCVEHBXTCB	
			1163.8	3	XPACNYMXSGPYYNXYTYDCVEHBXTCB	
			1092.0	4	XPACNYMXSGPYYNXYTYDCVEHBXTCBDNNDECB	
			959.0	5	XHDDCYGEAEBXPACNYMXSGPYYNXYTYDCVEHBXTCB	
			1198.5	4	XHDDCYGEAEBXPACNYMXSGPYYNXYTYDCVEHBXTCB	
10	0.33	16.0	561.8	2	ENYNXDXNB	PLA_2_; *H. hardwickii*; Q8UW31; Hcu|52837
			644.3	2	NMXBCANHGSR	
			435.2	3	NMXBCANHGSR	
			652.3	2	NMXBCANHGSR	
			668.8	2	XHDDCYGEAEB	
			692.3	2	TAAXCFAGAPYNB	
			516.6	3	ENYNXDXNBHCB	
			774.3	2	ENYNXDXNBHCB	
			644.3	3	DNNDECBAFXCNCDR	
			965.9	2	DNNDECBAFXCNCDR	
			829.7	3	TAAXCFAGAPYNBENYNXDXNB	
			728.8	4	TAAXCFAGAPYNBENYNXDXNBHCB	
			971.4	3	TAAXCFAGAPYNBENYNXDXNBHCB	
			983.7	3	MTXDYMDYGCYCGTGGSGTPVDEXDR	
			989.0	3	MTXDYMDYGCYCGTGGSGTPVDEXDR	
			1475.0	2	MTXDYMDYGCYCGTGGSGTPVDEXDR	
			1092.0	4	XPACNYMXSGPYYNXYTYDCVEHBXTCBDNNDECB	
			962.2	5	XHDDCYGEAEBXPACNYMXSGPYYNXYTYDCVEHBXTCB	
			1202.5	4	XHDDCYGEAEBXPACNYMXSGPYYNXYTYDCVEHBXTCB	
11	4.21	27.1	964.5	1	CBTEWXB	CRISP; *H. hardwickii*; Q8UW11; Hcu|52188
			1092.6	1	BCBTEWXB	
			1126.6	1	WNSHAABNAB	
			1194.7	1	EXVDBHNAXR	
			1342.6	1	CTFAHSPEHTR	
			1509.6	1	SBCPATCFCHNB	
			1776.8	1	YXYVCBYCPAGNXR	
	0.55	18.5	1342.6	1	CTFAHSPEHTR	CRISP; *H. hardwickii*; Q8UW11; Hcu|52188
			1776.8	1	YXYVCBYCPAGNXR	
	0.74	17.2	1342.6	1	CTFAHSPEHTR	CRISP; *H. hardwickii*; Q8UW11; Hcu|52570
	3.20	16.1	1776.8	1	YXYVCBYCPAGNXR	CRISP; *H. hardwickii*; Q8UW11; Hcu|52188

X: Leu/Ile; B: Lys/Gln. Methionine oxidation is underlined

Transcripts are listed in the Additional Table S2

*3-FTx* three finger toxin, *PLA*_*2*_ phospholipase A_2_, *CRISP* cysteine-rich secretory protein, *LNX* long chain α-neurotoxin; SNX, short chain α-neurotoxin

**Fig. 4:**
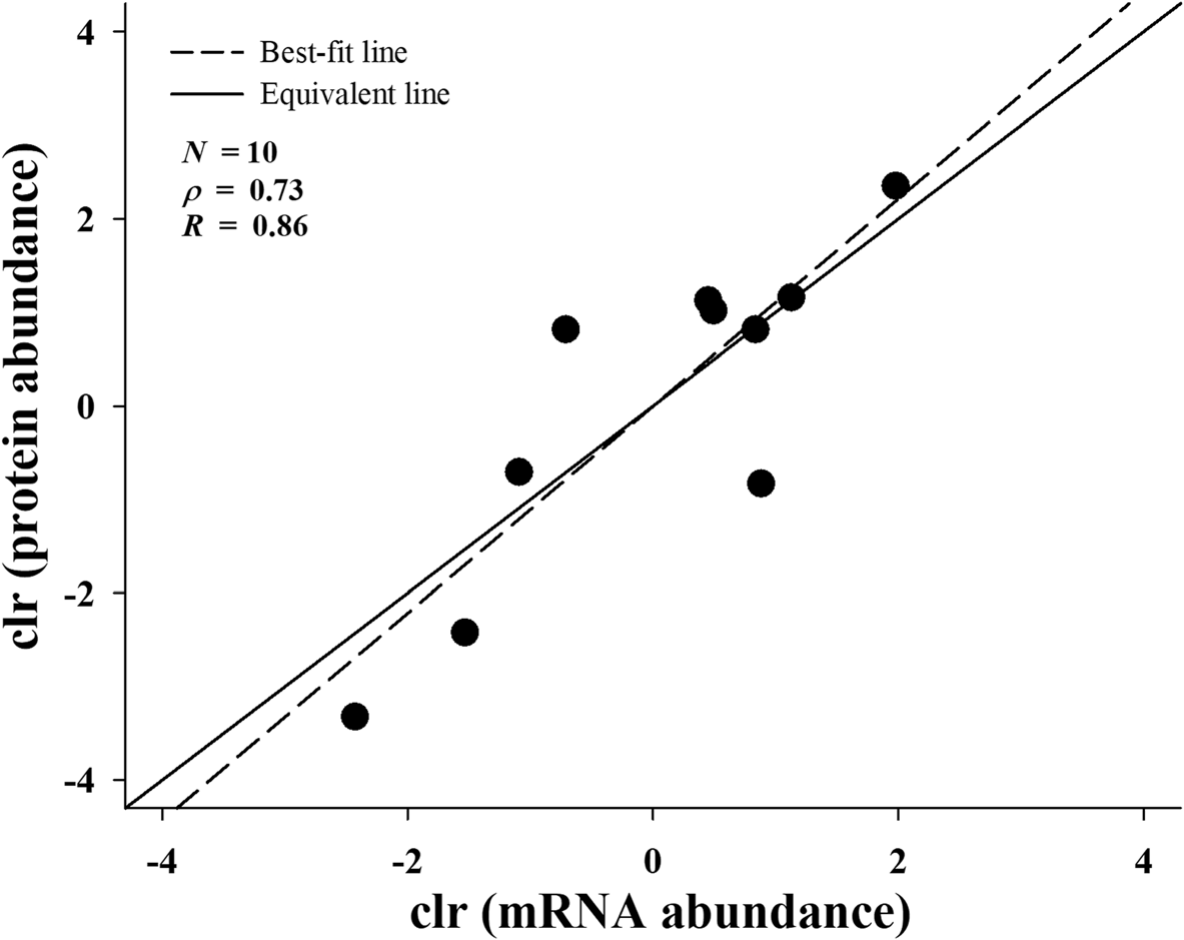
Correlation between mRNA and protein abundances of individual gene for each toxin family. SNX was excluded from the analysis. All data were centered log-ratio (clr) transformed. *N*, number of toxin transcripts; *ρ*, Spearman’s rank correlation coefficient; *R*, Pearson’s correlation coefficient

Moreover, as the proteins identified by MS could be assigned to 11 toxin-coding transcripts of three families (Table 1), the correlation between transcript and protein abundances of individual genes for each toxin family was further evaluated. Non-parametric correlation analysis revealed a weak correlation between both abundances of these 11 toxins with a low Spearman’s rank correlation coefficient (*ρ* = 0.30), whereas linear regression analysis indicated no correlation (*F*_1,9_ = 0.49, *P* = 0.51) with very low Pearson’s correlation coefficient (*R* = 0.23). In particular, extreme divergence between transcript and protein abundances could be observed for SNX (Fig. 4 and Table S1). When the analyses were conducted again by excluding SNX, a strong correlation between the abundances of the remaining 10 toxins was detected using non-parametric correlation analysis with *ρ* = 0.73 in addition to linear regression analysis (*F*_1,8_ = 21.8, *P* = 0.002) with *R* = 0.86.

### Adaptive molecular selection

Considering that not all full-length toxin-coding unigenes could be aligned with multiple homologous sequences with full CDS, we used either codeml or yn00 in PAML 4.8 to evaluate the potential for adaptive natural selection. Furthermore, one unigene (AMP) was excluded from the selection analysis because of the lack of homologs with full CDS. As excessive sequence divergence might reduce the power of the likelihood-ratio test [34], the unigenes from the same toxin family were divided into several groups at a threshold of 10% nucleotide sequence divergence and codeml analysis was conducted separately with their homologs. In total, 23 tests were performed using the codeml program on 15 toxin families including 30 full-length unigenes, with a significance level of 0.002 following Bonferroni correction (Table 2 and Additional Table S3). The selection of three full-length unigenes together with their homologs from the three toxin families was then directly analyzed using yn00.

**Table 2:** Summary of codeml tests for positive selection of toxins from venom-gland transcriptome in *H. curtus*

Toxins (No.)	M1: Nearly neutral			-ln*L*	M2: Positive selection				-ln*L*	M0: *ω*	Δ^a^	*P*-value^b^
3-FTx (1, 2, 3)	*p*:	0.28	0.72	748.17	*p*:	0.17	0.39	0.45	735.24	2.81	25.86	2.4 × 10^−6^*
	*ω*:	0.00	1.00		*ω*:	0.00	1.00	6.13				
3-FTx (4)	*p*:	0.26	0.74	804.61	*p*:	0.05	0.52	0.43	783.22	3.48	42.78	5.1 × 10^−10^*
	*ω*:	0.00	1.00		*ω*:	0.00	1.00	8.76				
3-FTx (5)	*p*:	0.51	0.49	652.06	*p*:	0.35	0.37	0.28	632.67	2.86	38.78	3.8 × 10^−9^*
	*ω*:	0.00	1.00		*ω*:	0.00	1.00	9.59				
CTL (1)	*p*:	0.30	0.70	843.02	*p*:	0.19	0.74	0.06	840.70	0.21	4.64	0.10
	*ω*:	0.00	1.00		*ω*:	0.00	1.00	24.33				
CTL (2)	*p*:	0.64	0.36	1221.13	*p*:	0.84	0.00	0.16	1212.49	0.58	17.28	1.8 × 10^−4^*
	*ω*:	0.05	1.00		*ω*:	0.22	1.00	3.50				
CTL (3)	*p*:	0.15	0.85	1160.65	*p*:	0.40	0.00	0.60	1148.75	2.43	23.80	6.8 × 10^−6^*
	*ω*:	0.00	1.00		*ω*:	0.17	1.00	4.52				
CTL (4)	*p*:	0.53	0.47	1201.51	*p*:	0.50	0.44	0.07	1196.01	0.65	11.00	0.004
	*ω*:	0.00	1.00		*ω*:	0.00	1.00	4.91				
PLA_2_ (1)	*p*:	0.49	0.51	978.57	*p*:	0.68	0.00	0.32	973.82	0.86	9.50	0.009
	*ω*:	0.00	1.00		*ω*:	0.13	1.00	3.17				
PLA_2_ (2, 3)	*p*:	0.44	0.56	967.20	*p*:	0.60	0.00	0.40	949.47	2.08	35.46	2.0 × 10^−8^*
	*ω*:	0.00	1.00		*ω*:	0.00	1.00	6.87				
SVMP (1)	*p*:	0.34	0.66	4316.80	*p*:	0.85	0.00	0.15	4292.41	1.08	48.78	2.6 × 10^−11^*
	*ω*:	0.00	1.00		*ω*:	0.57	1.00	5.53				
SVMP (2)	*p*:	0.51	0.49	7561.33	*p*:	0.35	0.47	0.18	7411.20	1.30	300.26	0.00*
	*ω*:	0.05	1.00		*ω*:	0.00	1.00	5.65				
SVSP (1)	*p*:	0.47	0.53	2042.88	*p*:	0.41	0.55	0.04	2028.38	0.73	29.00	5.0 × 10^−7^*
	*ω*:	0.01	1.00		*ω*:	0.00	1.00	7.78				
SVSP (2)	*p*:	0.46	0.54	3659.22	*p*:	0.30	0.47	0.23	3579.72	2.25	159.00	0.00*
	*ω*:	0.00	1.00		*ω*:	0.00	1.00	9.49				
5NT (1, 2)	*p*:	0.66	0.34	3064.74	*p*:	0.89	0.06	0.05	3057.77	0.40	13.94	9.4 × 10^−4^*
	*ω*:	0.00	1.00		*ω*:	0.19	1.00	6.10				
CRISP (1, 2)	*p*:	0.43	0.57	2473.69	*p*:	0.28	0.56	0.16	2428.77	1.63	89.84	0.00*
	*ω*:	0.00	1.00		*ω*:	0.00	1.00	6.69				
NGF (1, 2, 3)	*p*:	0.54	0.46	2549.71	*p*:	0.60	0.30	0.10	2527.86	0.86	43.70	3.2 × 10^−10^*
	*ω*:	0.12	1.00		*ω*:	0.28	1.00	4.35				
Cystatin	*p*:	0.49	0.51	823.89	*p*:	0.93	0.00	0.07	816.20	0.94	15.38	4.6 × 10^−4^*
	*ω*:	0.00	1.00		*ω*:	0.48	1.00	7.57				
HA	*p*:	0.52	0.48	3349.08	*p*:	0.56	0.00	0.44	3347.91	0.54	2.34	0.31
	*ω*:	0.00	1.00		*ω*:	0.00	1.00	1.28				
PDE	*p*:	0.54	0.46	5504.07	*p*:	0.57	0.31	0.12	5488.82	0.65	30.50	2.4 × 10^−7^*
	*ω*:	0.00	1.00		*ω*:	0.00	1.00	3.57				
PLA_2_ inhibitor	*p*:	0.49	0.51	1905.45	*p*:	0.50	0.00	0.49	1905.43	0.48	0.04	0.98
	*ω*:	0.00	1.00		*ω*:	0.00	1.00	1.06				
PLB	*p*:	0.55	0.45	4016.84	*p*:	0.51	0.46	0.02	4001.61	0.58	30.46	2.4 × 10^−7^*
	*ω*:	0.00	1.00		*ω*:	0.00	1.00	7.11				
QC	*p*:	0.77	0.23	2219.42	*p*:	0.78	0.00	0.22	2219.39	0.22	0.06	0.97
	*ω*:	0.00	1.00		*ω*:	0.00	1.00	1.08				
VEGF	*p*:	0.38	0.62	1221.70	*p*:	0.64	0.30	0.05	1214.99	0.82	13.42	1.2 × 10^−3^*
	*ω*:	0.00	1.00		*ω*:	0.43	1.00	7.44				

“*”, indicates significance at the 5% level after a Bonferroni correction

“^a^”, negative twice the difference in ln*L* between M1 and M2

“^b^”, *P*-value before correction

To analyze the 3-FTxs, five unigenes were first divided into three groups: 3-FTx(1, 2, 3), 3-FTx(4), and 3-FTx(5). The results indicated that the null hypothesis, the nearly neutral model (M1), could be easily rejected in favour of the positive selection model (M2) in the three tests, with all *P* < 0.001 following Bonferroni correction (Table 2). Moreover, 28–45% codon sites were found to have experienced positive selection with 6.13 ≤ *ω* ≤ 9.59. The single-ratio model (M0) indicated that all sites and branches of sequence experienced an average strength of selection with 2.81 ≤ *ω* ≤ 3.48.

The nucleotide sequence divergence among the four CTL unigenes was > 10%; therefore, these unigenes were analyzed separately with their homologs. In CTL(2) and CTL(3), M1 could be easily rejected in favour of M2, with all *P* < 0.01 following Bonferroni correction (Table 2). In addition, 16–60% codon sites experienced positive selection with 3.50 ≤ *ω* ≤ 4.12. However, M1 could not be rejected in CTL(1) and CTL(4) with *P* > 0.05 in all cases. M0 indicated that all sequence sites and branches experienced an average strength of selection with 0.21 ≤ *ω* ≤ 2.43.

In PLA_2_, the null hypothesis, M1, could only be rejected in one group, PLA_2_(2, 3), in favour of M2 with *P* < 0.001 following Bonferroni correction (Table 2). In addition, 40% of codon sites experienced positive selection with *ω* = 6.87. The null hypothesis could not be rejected in PLA_2_(1) with *P* = 0.21 following Bonferroni correction (Table 2). M0 showed that all sequence sites and branches experienced an average strength of selection of 0.86 ≤ *ω* ≤ 2.08.

Unigenes from both SVMP and SVSP families all exhibited > 30% divergence from one another; thus, they were analyzed separately along with their homologs using codeml. For all four tests, M1 could be readily rejected in favour of M2 with all *P* < 0.001 following Bonferroni correction (Table 2). Approximately 15–18% codon sites experienced positive selection with 5.53 ≤ *ω* ≤ 5.56 in the SVMP family, with estimated values in the SVSP family of 4–23% codon sites with 7.78 ≤ *ω* ≤ 9.49. M0 indicated that all sites and branches experienced an average strength of selection with 1.08 ≤ *ω* ≤ 1.30 in SVMP, whereas that estimated in the SVSP family was 0.73 ≤ *ω* ≤ 2.25.

The 5′NT, CRISP, and NGF families comprised 2–3 unigenes, with sequence divergence within each toxin family below 10%. Thus, the unigenes from the same family were combined for codeml analysis. The results indicated that the null hypothesis, M1, could be rejected in favour of M2 with all *P* < 0.05, following Bonferroni correction (Table 2). Additionally, 5% (5′NT), 16% (CRISP), and 10% (NGF) codon sites experienced positive selection with 4.35 ≤ *ω* ≤ 6.69. M0 showed that all sequence sites and branches experienced an average strength of selection with 0.40 ≤ *ω* ≤ 1.63.

Of the remaining families subjected to condeml tests, four (Cystatin, PDE, PLB, and VEGF families) easily rejected the null model, M1, and accepted M2 with *P* < 0.05 in all cases following Bonferroni correction (Table 2). For these four tests, an estimated 7, 12, 2, and 5% codon sites, respectively, experienced positive selection with 3.57 ≤ *ω* ≤ 7.57. M0 implied that all sites and branches of the four unigenes on average experienced a selection strength of 0.58 ≤ *ω* ≤ 0.94. Alternatively, tests for HA, PLA_2_ inhibitor, and QC unigenes could not reject the null hypothesis, M1, with all *P* > 0.05 following Bonferroni correction (Table 2); M0 indicated *ω* of 0.54, 0.48, and 0.22, respectively.

As only one homolog with high similarity could be obtained from the database, maximum-likelihood estimation based on *dN* and *dS* of the remaining three unigenes was conducted using the yn00 program. Comparing each pair of sequences, we estimated *dN* of 0.11, 0.04, and 0.003 for AchE (homologous sequence AB852000 from *Ovophis okinavensis*), CREGF (HQ414087 from *Crotalus adamanteus*), and MP inhibitor (MG132025 from *Bothrops moojeni*) of *H. curtus*, and *dS* of 0.18, 0.12, and 0.12, with a standard error < 0.04. Thus, we calculated *dN*/*dS* = 0.61, 0.31, and 0.02 for these three toxins, respectively, suggesting that these sequences probably experienced purifying selection.

### Antivenomic, ELISA, and western blotting evaluation of antivenom efficiency

**Fig. 5:**
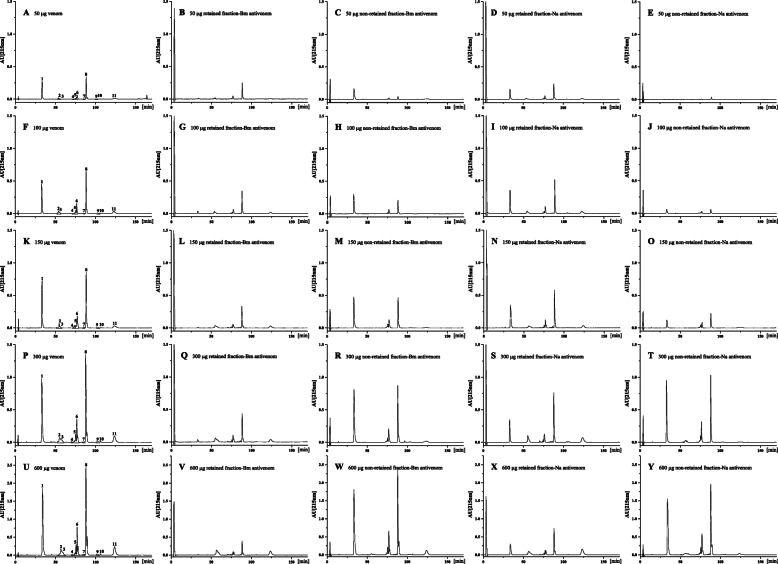
Antivenomics analysis of *H. curtus* venom using commercial antivenom by RP-HPLC. Chromatographic profiles of 50, 100, 150, 300 and 600 μg whole venom components (panels **A**, **F K**, **P** and **U**) and, of immunocaptured venom components (panels **B**, **D**, **G**, **I**, **L**, **N**, **Q**, **S**, **V** and **X**) and non-immunocaptured venom components (panels **C**, **E**, **H**, **J**, **M**, **O**, **R**, **T**, **W** and **Y**) recovered from the affinity columns after incubation with the corresponding amounts of venom. Bm, *B. multicinctus*; Na, *N. atra*

Using third-generation antivenomic analysis [35] to evaluate the efficacy of commercial monovalent *B. multicinctus* and *N. atra* antivenoms in capturing *H. curtus* venom components, we found that the capacity of *N. atra* antivenom to immunocapture the *H. curtus* venom was stronger than that of *B. multicinctus* antivenom, with increasing abundance of snake venom able to be captured by antivenom as the ratio of antivenom to venom increased (Fig. 5 and Table 3). Based on the ratio of antivenom (20 mg) to venom (50 μg) corresponding to approximately 48-fold molar excess per “10 kDa of toxins”, 53.5% of venom components, comprised mainly of long-neurotoxin (LNX), PLA_2_, and CRISP, could be immunocaptured by *B. multicinctus* antivenom, with the non-immunocaptured components including 94.3% of the SNX being minimally recognized. Comparatively speaking, 90.6% of the total venom components could be immunocaptured by *N. atra* antivenom (Fig. 5A-E and Table 3). However, when the cross-reaction was conducted between 600 μg venom and 20 mg antivenom with a ratio corresponding to approximately 4-fold molar excess per “10 kDa of toxins” (assuming an average molecular mass of 13 kDa for *H. curtus* venom components), the relative abundance of venom components immunocaptured by the antivenoms decreased dramatically. Specifically, only 17.7% of total venom components could be recognized by *B. multicinctus* antivenom with the abundance of non-immunocaptured components accounting for 99.1% SNX (peak 1), 84.8% PLA_2_ and long-neurotoxin (peak 5), 83.7% PLA_2_ (peak 6), 89.3% PLA_2_ (peak 8), and 50.4% CRISP (peak 11), respectively (Fig. 5U-W and Table 3). Similarly, only 27.9% of the total venom components could be recognized by *N. atra* antivenom, with the abundance of non-immunocaptured components accounting for 84.3% SNX (peak 1), 83.6% PLA_2_ and long-neurotoxin (peak 5), 75.3% PLA_2_ (peak 6), 76.8% PLA_2_ (peak 8), and 75.0% CRISP (peak 11), respectively (Fig. 5U, X, Y, and Table 3). In the other three reactions, the antivenoms could immunocapture 43.1% (100 μg), 35.0% (150 μg), and 30.6% (300 μg) of whole venom by *B. multicinctus* antivenom, and 83.0, 69.6, and 44.0% of that by *N. atra* antivenom (Fig. 5F-T and Table 3).

**Table 3:** Total and concentration-dependent immunoretained *H. curtus* venom proteins by heterologous antivenoms

Fractions		50	100	150	300	600	Toxins
1	μg Total	16.56	33.13	49.69	99.38	198.77	3-FTx (SNX)
	μg RET-Bm	0.95	2.94	1.18	4.17	1.82	
	μg RET-Na	16.56	26.48	34.36	25.62	31.13	
2	μg Total	2.31	4.61	6.92	13.84	27.69	3-FTx (LNX)
	μg RET-Bm	2.14	4.61	6.92	13.84	24.66	
	μg RET-Na	2.31	4.61	6.92	10.39	17.71	
3	μg Total	1.14	2.29	3.43	6.86	13.73	3-FTx (LNX)
	μg RET-Bm	1.14	2.26	3.43	6.86	12.40	
	μg RET-Na	1.14	2.29	3.43	5.37	8.16	
4	μg Total	0.17	0.34	0.50	1.01	2.01	3-FTx (LNX)
	μg RET-Bm	0.17	0.34	0.50	1.01	2.01	
	μg RET-Na	0.17	0.34	0.48	0.92	0.93	
5	μg Total	2.05	4.10	6.15	12.3	24.6	PLA_2_ + 3-FTx (LNX)
	μg RET-Bm	0.81	1.38	1.53	4.03	3.73	
	μg RET-Na	1.04	2.16	2.15	4.03	4.02	
6	μg Total	4.95	9.90	14.84	29.69	59.37	PLA_2_
	μg RET-Bm	3.44	4.42	5.48	11.17	9.69	
	μg RET-Na	4.44	7.41	8.59	11.71	14.67	
7	μg Total	0.43	0.85	1.28	2.56	5.12	PLA_2_ + 3-FTx (LNX)
	μg RET-Bm	0.43	0.85	1.28	2.56	1.71	
	μg RET-Na	0.43	0.85	1.28	2.56	1.24	
8	μg Total	17.48	34.96	52.44	104.87	209.75	PLA_2_
	μg RET-Bm	14.79	20.02	21.4	31.55	22.51	
	μg RET-Na	15.03	30.25	35.92	44.18	48.59	
9	μg Total	0.40	0.79	1.19	2.38	4.77	PLA_2_
	μg RET-Bm	0.13	0.23	0.47	0.92	0.46	
	μg RET-Na	0.40	0.56	0.69	1.73	1.01	
10	μg Total	0.16	0.33	0.49	0.99	1.98	PLA_2_
	μg RET-Bm	0.11	0.33	0.20	0.87	0.62	
	μg RET-Na	0.16	0.33	0.42	0.99	0.69	
11	μg Total	4.35	8.70	13.05	26.11	52.22	CRISP
	μg RET-Bm	2.63	5.74	10.06	14.72	26.34	
	μg RET-Na	3.59	7.66	10.13	24.57	39.14	

*RET* immunoretained, *Bm B. multicintus* antivenom, *Na N. atra* antivenom

We also found that the capacity of the antivenoms to immunocapture the peak fractions differed and that the immunocapture efficiency changed nonsynchronously as the amount of venom increased (Fig. 5 and Table 3). Additionally, the maximal immunocapturing capacity of *N. atra* antivenom for peaks 1, 6, and 8–11 was higher than that of *B. multicinctus* antivenom, whereas the effects were opposite for peaks 2–4 and comparable for peaks 5 and 7. It could be calculated that 20 mg of immobilized *B. multicinctus* and *N. atra* antivenom presented a maximal immunocapturing capacity of 120.7 and 172.9 μg of *H. curtus* venom, respectively.

**Fig. 6:**
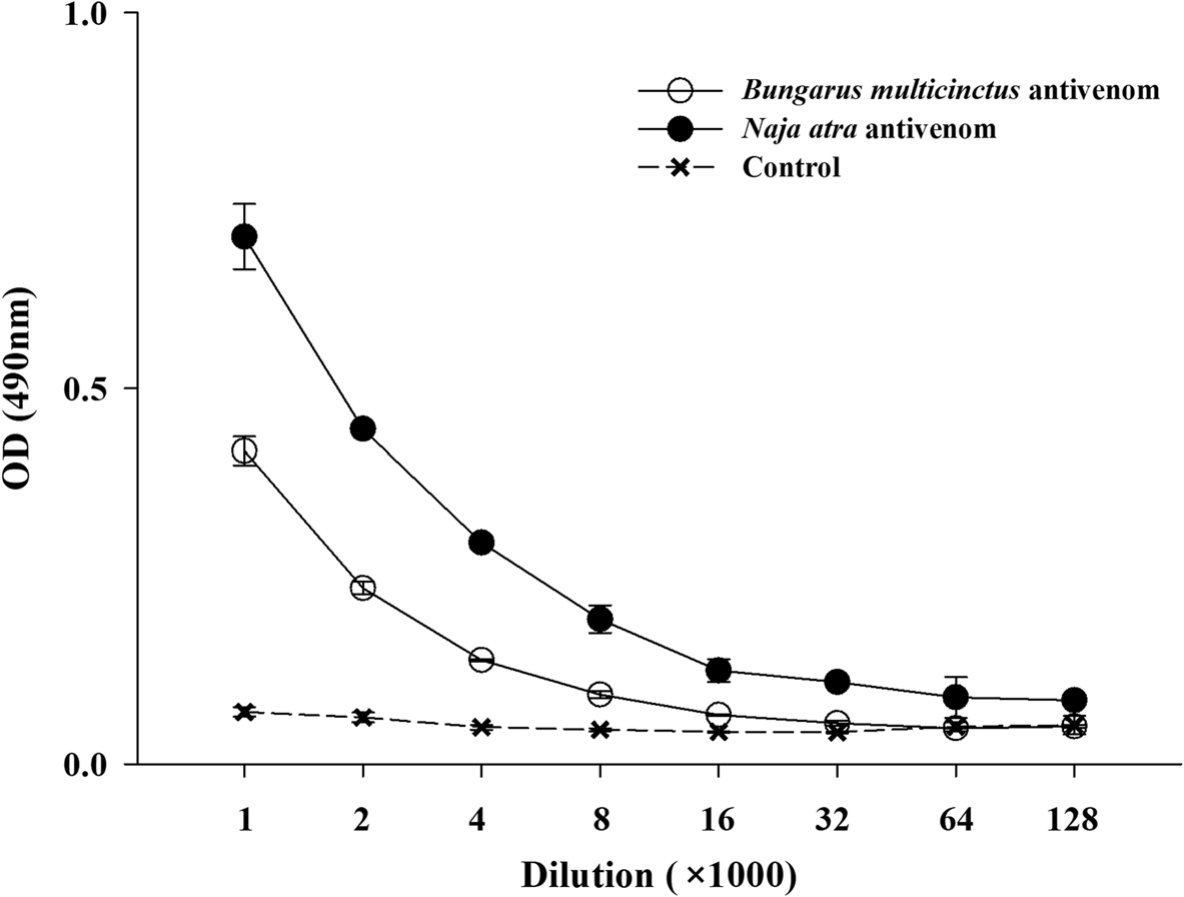
Cross-reaction between *H. curtus* venom and commercial antivenom determined by ELISA. Normal horse serum was used as negative control. Data are expressed as mean ± SD (*n* = 3)

**Fig. 7:**
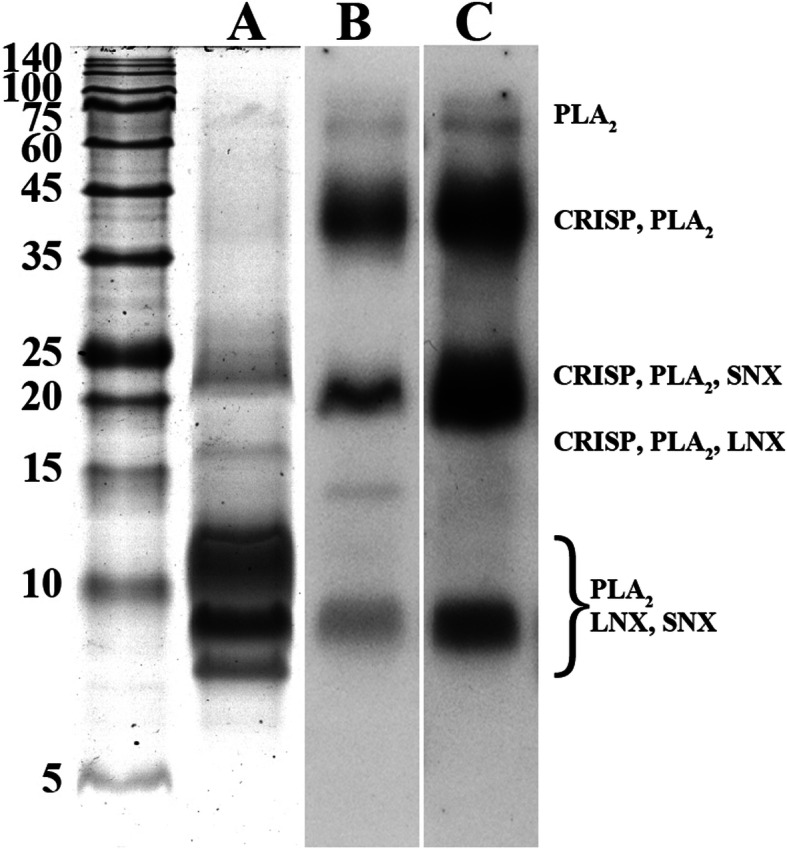
Cross-reaction between *H. curtus* venom and commercial antivenom determined by western blotting. **A** SDS-PAGE profiles of venom protein (identified proteins were listed in Table S4); **B** *B. multicinctus* antivenom; **C** *N. atra* antivenom (original images were listed in Figure S2). PLA_2_, phospholipase A_2_; CRISP, cysteine-rich secretory protein; LNX, long chain α-neurotoxin; SNX, short chain α-neurotoxin

A significant cross-reaction between *B. multicinctus*/*N. atra* antivenom and *H. curtus* venom could be detected by ELISA analysis, with the cross-reactivity showing a progressive increase as the concentration of antivenom increased; moreover, the *N. atra* antivenom presented relatively higher cross-reactivity than that of the *B. multicinctus* antivenom (Fig. 6). A similar capacity was demonstrated using western blotting, another conventional protocol that is commonly used to assess the preclinical efficacy of antivenom. The electrophoretic profile revealed that five significant bands with molecular masses ranging from 8 to 21 kDa could be detected in *H. curtus* venom, of which three (8, 9, and 11 kDa) were present high abundance in venom whereas two bands with molecular masses of 16 and 21 kDa were expressed at relatively low abundance; and another two bands with relatively higher molecular masses of 40 and 60 kDa were expressed at extremely low abundance (Fig. 7A). Mass spectrometry analysis indicated that six protein bands are comprised of more than one protein family except the one with molecular mass of 60 kDa (Fig. 7 and Additional Table S4). According to the peptide intensity, one group of protein bands (8 and 11 kDa) were mainly comprised of relatively high abundance of LNX and SNX, and the other two groups (9, 16 and 60 kDa; 21 and 40 kDa) mainly contained PLA_2_ and CRISP, respectively (Additional Table S4). Protein bands with molecular masses higher than 21 kDa were strongly recognized by *B. multicinctus* and *N. atra* antivenoms but only one (9 kDa) of the three protein bands with the lowest molecular mass could be recognized by these two antivenoms (Fig. 7B and C). The cross-reaction was mainly located in the protein bands with molecular masses of 9, 21, and 40 kDa, with *N. atra* antivenom affording a higher cross-reaction intensity than that from *B. multicinctus* antivenom (Fig. 7B and C). Overall, these results indicated that *N. atra* antivenom presented a higher capacity to immunocapture *H. curtus* venom than *B. multicinctus* antivenom and, both antivenoms displayed relatively higher capacity to immunocapture CRISP and PLA_2_ than that to 3-FTx (LNX and SNX).

## Discussion

Knowledge of venom-gland transcriptomic and venomic profiles is generally considered to be useful in elucidating the evolutionary process and potential function of snake venom, developing antivenom, and even exploring venom-based drugs [36–42]. Notably, the maturation and widespread application of high throughput sequencing and MS technologies have rendered it much easier to unravel the complexity of snake venoms worldwide at the -omics level. In the present study on *H. curtus* from the South China Sea, 22 and 3 toxin families were identified in the venom-gland transcriptome and venom proteome, respectively, using next-generation sequencing technology and a combined proteomic strategy.

Notably, the composition and abundance of *H. curtus* toxins identified in the present study apparently differed from those in previous investigations either at the mRNA or protein level [6, 8, 19]. For example, the toxin transcripts of the *H. curtus* venom gland was shown to be mainly comprised of 3-FTx, PLA_2_, CRISP, and PDGF families using Sanger sequencing, with the first three families accounting for 55.5% of the total venom-gland transcriptome and the 3-FTx family expressed more abundantly than PLA_2_ at a ratio of 4.4:1 [19]. Comparatively speaking, although 3-FTx, PLA_2_, and CRISP transcripts also constituted the most abundant components in the venom-gland transcriptome of *H. curtus* in the present study, accounting for 99.68% of total toxin transcripts, 3-FTx was less abundant than PLA_2_ at a ratio of 1:2.2. Moreover, 19 toxin families expressed with low abundance (0.32%) among total toxin transcripts were also detected in our sample. Next-generation sequencing technology therefore appeared to be more powerful than Sanger sequencing for detecting the snake venom-gland transcripts with low abundance. Moreover, the venom protein/mRNA sequence database was relatively insufficient 10 years ago, such that a high proportion (24.3%) of transcripts in a previous study could not be matched to the targeted toxins, potentially resulting in additional toxin families being omitted.

When compared with previously investigated *H. curtus* venoms from Australia and Malaysia [6, 8], the venom from the South China Sea population evaluated in the presented study diverged significantly with regard to the relative abundance of predominant toxins at the protein level. The South China Sea population exhibited the lowest abundance of PLA_2_ but the highest abundance of 3-FTx, especially SNX, which was approximately 4- and 1.45-fold higher than those from Australia and Malaysia, respectively. Notably, given that SNX was determined to be the most toxic component in *H. curtus* venom [6], the South China Sea population might possess the highest venomic lethality among the three localities.

Moreover, although the venom-gland transcriptome and venom proteome of *H. curtus* were dominated by 3-FTx, PLA_2_, and CRISP families, of which the total abundance expressed at the protein level was highly consistent with that at the mRNA level, an apparent discrepancy was still observed with regard to the composition and abundance of toxins at the family level. Given that comparisons at the family level may not necessarily reflect the mechanisms affecting specific toxin transcripts, a detailed analysis of the correlation between mRNA and protein abundances of 11 toxins for 3-FTx, PLA_2_, and CRISP families was conducted, with the results agreeing with those at the family level. Such discordance between both levels has been observed in many venomous snakes, being potentially attributed to transcriptional, post-transcriptional, translational, or post-translational regulatory mechanisms [11–13, 43–46] in addition to the time span between collection of the venom and venom-gland tissue [14]. In contrast, some studies revealed little differential expression of toxins at both -omics levels despite individual variation [18, 47, 48]. Notably, a strong correlation between both mRNA and protein levels was detected in the present study in the remaining toxin transcripts when SNX was excluded. Thus, the mechanisms regulating the mRNA and protein abundances of each toxin from *H. curtus* may differ. Nevertheless, as the majority of *H. curtus* toxins could only be detected at the mRNA but not the protein level, it remains to be explored whether this phenomenon is regulated by more complex mechanisms such as post-transcriptional silencing and protein buffering.

Considered as a highly effective and biochemically complex weapon involved in predation, digestion, and defense, snake venom is generally considered to have undergone positive selection during adaptive evolution, which has been successfully estimated at individual toxin and venom-gland transcriptomic levels [49–56]. To deal with diverse preys, a relatively high proportion of toxins appears to experience positive selection at transcriptomic level in many terrestrial venomous snakes, such as 24 of 27 full-length toxin-coding transcripts from venom-gland transcriptome were detected to be underwent positive selection in *Crotalus adamanteus* [52] and, nearly all of the venom-gland toxin ESTs had sites that clearly showed evidence of positive selection signals in *B. multicinctus* and *N. atra* [56]. Consistent with the results of these studies, the majority of the toxin transcripts (24/33) from *H. curtus*, accounting for 73% of the total analyzed transcripts, were deemed in the present study to have experienced positive selection according to the likelihood ratio from M1 and M2; and it was also verified by M7 and M8 (the results were not shown in this paper as they nearly identical to that from M1 and M2). That is to say the adaptive evolution of toxin genes from *H. curtus* can still be extensively driven by natural positive selection, even as the diet of *H. curtus* are simpler than those of its terrestrial relatives. Some studies proposed that toxin genes experience both stronger positive selection and lower selective constraint than non-toxin genes, and toxins possessed with the predominant physiological functions and expressed relatively high abundance evolve more rapidly due to the positive selection [53, 57, 58]. Here, it could be easily observed that the average *dN*/*dS* of 3-FTx, PLA_2_ and CRISP families are relatively larger than that of the other toxin families in *H. curtus*. It is thus confirmed that the force strength of positive selection is similarly stronger in those *H. curtus* venom toxins expressed important physiological functions and higher abundance, which might imply a potential interplay between abundance and adaptive evolution of toxins. Furthermore, the rapidly adaptive evolution driven by positive selection in toxins might be accompanied with mutations in vital nucleotide sites and the followed reduction (even loss) of toxin efficiency, while purifying selection would probably aid in preserving the venom potency by filtering out these mutations with deleterious effects [59]. It is thus believed that the different strength of purifying selection in *H. curtus* toxins will provide evolutionary implications in optimizing the trade-off between venom potency and adaption to a simplified diet.

The antivenomic analysis approach based on venomic profile, originally developed as an in vitro platform to qualitatively and quantitatively assess the efficacy of antivenom, has since been updated to the 3rd generation and displays a powerful potential as a substitute for many traditional strategies to evaluate antivenom efficacy [35]. Similar to several recently reported studies using 3rd generation antivenomics [60–62], the immunocapture capacity of antivenoms in the present study increased as the incubation amount of *H. curtus* venom increased but varied according to different venom components. Overall, *N. atra* antivenom exhibited higher capacity to immunocapture whole *H. curtus* venom than *B. multicinctus* antivenom, especially for SNX, PLA_2_, and CRISP. However, the former demonstrated a relatively lower capability to immunocapture the LNX. This suggests that *N. atra* antivenom possesses a greater abundance of F(ab′)_2_ to efficiently recognize *H. curtus* venom than *B. multicinctus* antivenom at the same dose, but the amounts of F(ab′)_2_ to recognize specific venom components are likely different between these two antivenoms. This divergence was also roughly verified by western blotting and ELISA analyses. Given that six of seven protein bands from *H. curtus* venom directly separated by SDS-PAGE were comprised of more than one protein family, western blotting analysis displayed weak ability to qualitatively and quantitatively evaluate the capacity of both antivenoms to recognize each venom component. Although it was found both antivenoms cannot efficiently recognized most of 3-FTx in *H. curtus* venom by 3rd generation antivenomic and western blotting analyses, it was still hard to associate the results from both two analyses precisely. Briefly, western blotting analysis only roughly indicated that the capacity of both antivenoms to recognize the venom components with low molecular mass (≤ 15 kDa, mainly 3-FTx and PLA_2_) was relatively weaker than that for recognizing high molecular mass components (mainly CRISP and PLA_2_), with no ability to recognize the components (mainly 3-FTx) with molecular masses of 8 and 11 kDa. This might be due to the lack of homologous components between *B. multicinctus*/*N. atra* and *H. curtus* venoms, or because 3-FTx generally induce limited or no antibodies because of their weak immune response.

Given the high similarity in venom composition among closely related snakes, appropriate heterologous antivenoms to treat envenomations caused by snakes with no commercial antivenom might be selected according to the phylogenetic relationship. For example, patients envenomed by sea snakes including *H. curtus* have been often been treated by *B. multicinctus* and *N. atra* antivenoms in some hospitals in China [31]. However, based on the antivenomic profiles generated in the present study, *N. atra* antivenom appears to be more suitable for treatment of patients envenomed by *H. curtus* than *B. multicinctus* antivenom, although the phylogenetic relationship between *H. curtus* and *B. multicinctus* is much closer than that between *H. curtus* and *N. atra* [63]. Thus, the phylogenetic relationship should not be a unique criterion for selection of alternative antivenoms in clinic treatment of snakebites.

Specifically, based on the maximal immunocapture capacity, one vial of commercial *B. multicinctus* (approximately 57 mg/mL, 10 mL/vial) and *N. atra* (124 mg/mL, 10 mL/vial) antivenoms theoretically could be inferred to absorb 3.4 and 10.7 mg *H. curtus* venom components, respectively, which appeared to approximate 28 and 89% of the average venom yield of 12.0 mg (lyophilized venom, calculated from six specimens in the present study). However, it should be noted that the maximal amount of venom components immunocaptured by the antivenoms may not be equal to that of whole venom; e.g., 20 mg *N. atra* antivenom could maximally absorb 172.9 μg venom components but only absorbed 69.6% of 150 μg whole *H. curtus* venom. Moreover, both antivenoms only exhibited high immunocapture efficiency when incubated with relatively low amounts of venom, with up to 53.5 and 90.6% of the total amount of 50 μg venom being absorbed by *B. multicinctus* and *N. atra* antivenoms, respectively. The abundance of unabsorbed venom components increased as the ratio of antivenom to venom decreased, especially for SNX, the most toxic component in *H. curtus* venom [6]. Consistent with this, the clinical efficacy of both *B. multicinctus* and *N. atra* antivenoms in the treatment of sea snake envenomations has also been relatively poor [31]. Accordingly, the development and application of species-specific antivenom against *H. curtus* venom in China is urgently needed.

## Conclusion

An integrated omics-strategy was used to reveal the venom-gland transcriptomic, venomic, and antivenomic profiles of *H. curtus* from the South China Sea. Our findings verify discordance between the venom-gland transcriptome and venom proteome in *H. curtus*. Briefly, the venom evolved to as a ‘biochemically simple’ weapon at the protein level to adapt to the simplified diet available when the sea snake returned to the marine environment; conversely, the toxin transcripts with high diversity in the venom-gland transcriptome of *H. curtus* could be defined as a ‘genetically complicated’ mixture at the mRNA level. In addition, the majority of the full-length toxin-coding unigenes may have experienced positive selection in the evolutionary history. However, it should not be ignored that the transcripts of our sample have not been assembled based on a targeted reference genome, which might potentially hamper the identification of toxins that are not detected in venom-gland transcriptome and proteome and, exploration of more insights into toxin evolution including gene duplication and pseudogenization, etc. Moreover, *N. atra* antivenom has a stronger capability to immunocapture *H. curtus* venom components than does *B. multicinctus* antivenom, but both antivenoms only exhibit high immunocapture efficiency when they are incubated with relatively low amounts of venom. Thus, the clinical application of commercial *N. atra* and *B. multicinctus* antivenoms is not recommended in treatment of patients envenomed by *H. curtus*. Rather, the development and application of species-specific antivenom of *H. curtus* venom is urgently required in China. Taken together, our results provide a foundation for additional studies of population-specific venom molecular and evolutionary analyses and the development of effective *H. curtus* antivenom, and the antivenomic strategy based on venomic profile for qualitatively and quantitatively evaluating the efficiency of antivenom would present a well development future.

## Methods

### Venom and antivenoms

We obtained six adult specimens of *H. curtus* as by-catch in the South China Sea and transported them to our laboratory at Hainan Tropical Ocean University, and maintained them in a circulating sea water system for venom extraction. Fresh venom from individual snakes was extracted repeatedly at a 15-day interval using a 100-μl plastic pipette, centrifuged to remove impurities for 15 min at 10,000 g 4 °C, lyophilized, pooled equally, and stored at − 80 °C until use. Commercial monovalent *B. multicinctus* antivenom (batch 20,070,601, expiry date: 06/2010) and *N. atra* antivenom (batch 20,070,601, expiry date: 06/2010) consisting of purified F(ab′)_2_ fragments from the plasma of hyperimmunized horses were purchased from Shanghai Serum Biological Technology Co., Ltd., China. Both antivenoms were subdivided, lyophilized, and stored at − 80 °C immediately upon receipt in the laboratory. Snakes were collected under the permit (2017-09-12) issued by Hainan Tropical Ocean University, and our experimental procedures was approved by the Animal Research Ethics Committee of Hangzhou Normal University (AREC2019109).

### Venom gland cDNA synthesis and sequencing

The snakes were sacrificed for venom-gland transcriptomic analysis when sufficient crude venom was accumulated. After the last round of venom extraction, the snakes were allowed to recover for 4 days to maximize the venom gland transcription and then anesthetized heavily with sodium pentobarbital (s.c. 30 mg/kg) until they showed no tail-retraction reflex. Two venom glands of each snake were removed and split into pieces with a diameter of < 2 mm, then pooled and permeated with RNAlater® (Qiagen) at 4 °C overnight before transferring to − 80 °C storage until use. The snakes then were euthanized by injection of sodium pentobarbital (i.p. 100 mg/kg), and the carcasses were preserved with 10% formalin and deposited in the animal collections of Hainan Tropical Ocean University. Total RNA of the pooled venom gland tissue from each sample was isolated using TRIzol (Life Technologies) according to the manufacturer’s instructions. RNA was purified, concentrated, and then resuspended in 100 μl THE Ambion® RNA storage solution (Life Technologies). The degree of contamination and degradation of RNA in each sample was monitored by 1% agarose gel electrophoresis. The purity and integrity of RNA was assessed using a Implen NanoPhotometer (Implen GmbH) and Agilent 2100 Bioanalyzer system (Agilent Technologies), respectively, whereas the concentration of RNA was measured using a Qubit 2.0 fluorometer (ThermoFisher Scientific). Subsequently, an RNA mixture for sequencing was prepared by mixing equal amounts of RNA from the above six samples.

The cDNA library for RNA sequencing was generated using the NEBNext® Ultra™ RNA Library Prep Kit for Illumina® (New England Biolabs) according to the manufacturer’s instructions. In brief, mRNA was purified and enriched from total RNA with oligo (dT)-attached magnetic beads. Then, the mRNA was fragmented and used as a template to synthesize first-strand cDNA with reverse transcriptase (RNase H) and random hexamer primers. The following step for synthesizing second-strand cDNA was conducted with dNTPs, RNase H and DNA polymerase I based on first-strand cDNA. The resultant double-stranded cDNA was purified using AMPure XP beads (Beckman Coulter) and subjected to the processes of end repair and ligation of a poly (A) tail and adapters. The adapter-ligated fragments were preferentially screened for those at 250–300 bp in length, amplified by PCR, and purified using AMPure XP beads to create the final cDNA library. Quality of the library was assessed using the Agilent 2100 Bioanalyzer system (Agilent Technologies). High throughout sequencing of the cDNA library was performed using the Illumina HiSeq™2500 platform at Novogene Bioinformatics Technology Co., Ltd., China (www.novogene.cn).

### Transcriptome assembly, annotation, and quantification

Prior to transcriptome assembly, raw reads (raw data) from Illumina sequencing were cleaned by eliminating the reads containing adapters and > 10% unknown sequences (‘N’s), as well as the reads with low quality (Q ≤ 20). The clean reads (remaining reads) were assembled into contigs using Trinity 2.4.0 based on Inchworm, Chrysalis, and Butterfly modules [64]. Initially, clean reads were grouped into different read sets and assembled into a k-mer dictionary (*k* = 25), which was then developed into a collection of linear contigs by greedily searching using Inchworm. If the contigs shared at least one *k* – 1-mer and the reads spanned the junction between contigs, they were pooled and built into *de Bruijn* graphs using Chrysalis. Finally, the *de Bruijn* graphs with clean reads and paired-end reads were reconciled and the full-length transcripts for spliced isoforms and paralogous sequences were reconstructed and arranged using Butterfly. Subsequently, the longest sequence with no redundancy was generated from the transcripts in each gene locus by Corset 1.05 and defined as a unigene, which was used as a reference for downstream analyses. Gene annotation was performed by BLAST 2.2.28 searching against the NCBI NT database and Diamond 0.8.22 searching against the NCBI NR and UniProt protein databases (strict to the taxa Serpentes). The E-value threshold was set to 1 × 10^− 5^. To estimate the abundance of unigenes, all clean reads were initially matched with the reference unigenes using RSEM 1.2.15 [65]. The number of reads matched to a given unigene was defined as the readcount, which was then calculated as FPKM [66] for evaluating the abundance.

### Isolation and characterization of venom proteins

Venom powder (3 mg) collected from the six snakes was reconstituted in 0.1% TFA and centrifuged for 15 min at 10,000 g, 4 °C. The supernatant was collected and applied to a Kromasil 300 RP-C18 column (250 × 4.6 mm, 5 μm; AkzoNobel) using a Waters E600 HPLC system (Waters). The venom proteins were separated at a flow rate of 1 ml/min with a mobile phase system of 0.1% TFA in water (solution A) and 100% ACN (solution B) at the following linear gradient: 0–15% B for 30 min, followed by 15–45% B for 120 min and 45–70% B for 20 min. Protein detection was conducted at 215 nm. Fractions were collected and concentrated in a CentriVap® Centrifugal Concentrator (Labconco). Protein concentrations of venom fractions were determined according to the Bradford method [67]. The proteins in each fraction were separated by 18% SDS-PAGE. The gels were stained in 0.2% CBB R-250 and scanned using a UMAx2100 densitometer (Novax Technologies).

Protein bands in the gels were excised, destained with 0.1 M NH_4_HCO_3_ in 30% ACN, rinsed with 0.1 M NH_4_HCO_3_, and then digested with trypsin (Promega) for 20 h at 37 °C. The peptide mixture was lyophilized, and re-dissolved in 2 μl of 20% ACN. The solution was spotted onto a sample holder and air-dried, to which 0.5 μl of 5 mg/ml α-cyano-4-hydroxycinnamic acid (ABI) in 50% ACN and 0.1% TFA was added; then, the sample was dried completely and subjected to 4800 Plus MALDI-TOF/TOF-MS mass spectrometer (AB SCIEX) according to the instruction manual. For LC-MS/MS analysis, the peptide mixture was re-dissolved in 0.1% TFA and desalted using a Zorbax 300 SB-C18 column (150 × 0.3 mm, 5 μm; Agilent Technologies), then separated by reverse phase capillary HPLC using an RP-C18 column (150 × 0.15 mm, 5 μm; Column Technology Inc.) with a mobile phase system of 0.1% FA in water (solution A) and 84% ACN in 0.1% FA (solution B) as follows: 4–50% B for 30 min, followed by 50–100% B for 4 min and 100% B for 1 min. The eluted peptides were applied to a Q Exactive mass spectrometer (ThermoFisher Scientific) according to the manufacturer’s instructions. The raw MS/MS spectra were interpreted using FlexAnalysis 3.4 or Xcalibur 2.2 software and the assignment of amino acid sequence similarity was executed using PEAKS X against the UniProt Serpentes database or an in-house database constructed using toxin transcripts extracted from the *H. curtus* venom-gland transcriptome. The mass tolerance was set at 0.4 Da for MALDI-TOF/TOF-MS and 0.1 Da for LC-MS/MS. Carbamidomethyl (C) was set as a fixed modification and oxidation (M) was set as a variable modification.

Integration of the collected HPLC fractions and densitometry of the protein bands in the SDS-PAGE electropherogram were used to estimate the relative abundance of venom composition according to Calvete et al. [68] and Shan et al. [69]. Briefly, the relative abundance of fractions was calculated by peak area measurement using Empower 2.0 software. When the fractions presented only one protein band in SDS-PAGE, the relative abundance was directly obtained from the peak area measurement, whereas for the fractions presenting more than one protein band, the relative abundance of each band was estimated by densitometry using Tan4100 software.

### Detection of adaptive molecular evolution

Tests of adaptive molecular evolution were performed on the transcripts with full-length CDS from 18 toxin families using positive selection analysis. Prior to analysis, sequences homologous to transcripts were obtained from the NCBI NT database with an approximate threshold of 10% nucleotide sequence divergence. Sequences were aligned using Geneious 4.8.3 on the basis of the amino-acid sequence. Gaps, stop codons, and signal peptides were excluded from all analyses. The best-fitting model for partitions was determined using PartitionFinder 1.1.1 [70] based on the Bayesian Information Criterion and greedy search. Then, a Bayesian phylogeny was constructed using BEAST 2.2 [71] and each analysis was conducted by performing four replicate searches with 100 million generations, retaining trees every 10,000 generations. The chains were ensured to converge and mix adequately using Tracer 2.2. The maximum clade credibility tree for the target tree was obtained using the TreeAnnotator 2.2 suite, with the first 10% of sampled generations being excluded.

A likelihood-ratio test for positive selection was performed using codeml in PAML 4.8, based on five models [72] as follows. The nearly neutral model (M1), which is defined as the null model, allows for a group of sites to have experienced neutral selection (*dN*/*dS* = 1) and another group to be constrained under purifying selection (*dN*/*dS* < 1) in evolutionary history. The positive model (M2), which is defined as the alternative model, advances an additional parameter that can indicate the group of sites that experienced positive selection (*dN*/*dS* > 1). To test whether a group of sites experienced positive selection, the difference in log likelihoods between these two models was compared to a χ^2^ distribution with two degrees of freedom. A similar test by comparing models M7 (beta) and M8 (beta with positive selection) was conducted to verify the initial results, using a χ^2^ distribution with two degrees of freedom. An overall *dN*/*dS* was indicated by the M0 model, which introduces a single ratio for all sites based on an average *dN*/*dS* across the entire phylogeny. If the transcript could only be aligned with one full-length homolog from the database, then *dN* and *dS* were directly analyzed by yn00 in PAML 4.8, and the *dN*/*dS* value for selection assessment was calculated manually.

### Antivenomics analysis

The capability of commercial monovalent *B. multicinctus* antivenom and *N. atra* antivenom to recognize *H. curtus* venom was assessed using the third-generation antivenomics analysis [35]. The antivenom was dissolved and dialyzed against MilliQ water, then lyophilized and re-dissolved in coupling buffer (0.2 M NaHCO_3_, 0.5 M NaCl, pH 8.3). An immunoaffinity column coupled with *B. multicinctus* or *N. atra* antivenom was prepared simultaneously. CNBr-activated Sepharose 4B matrix (1 ml) (GE Healthcare) was packed in a 3 ml column and washed with 15 CV (matrix volumes) of 1 mM ice-cold HCl, followed by 3 CV of coupling buffer. The matrix was incubated overnight at 4 °C in 0.5 CV of coupling buffer containing 70 mg antivenom. To calculate the antivenom coupling yield, the concentrations of antivenom before and after incubation with the matrix were determined spectrophotometrically at 280 nm based on an extinction coefficient (ε) of 1.36 for 1 mg/ml protein in a 1 cm light path length. The matrices of the two columns were coupled with 20.1 mg *B. multicinctus* and 20.5 mg *N. atra* F(ab′)_2_ antivenom fragments. After the coupling, the non-reacting groups were blocked gently with 1 CV of blocking buffer (0.1 M Tris-HCl, pH 8.0) for 4 h at room temperature on an orbital shaker. Both columns containing matrix were alternately washed with six repetitions of 3 CV of low (0.1 M acetate buffer, 0.5 M NaCl, pH 4.0) and high (0.1 M Tris-HCl, pH 8.5) pH buffer and equilibrated with 3 CV of binding buffer (20 mM PBS, pH 7.2). Finally, the matrix coupled with 20 mg of antivenom was retained in each column. For immunoaffinity analysis, 0.5 ml binding buffer containing 50, 100, 150, 300, and 600 μg *H. curtus* venom was loaded onto the column and incubated gently for 1 h at room temperature on an orbital shaker. After recovering the non-retained venom fractions using 3 CV binding buffer, the retained fractions were eluted and collected with 3 CV of 0.1 M glycine-HCl, pH 2.0, and immediately brought to neutral pH with 1 M Tris-HCl (pH 9.0). Both non-retained and retained fractions were concentrated using a CentriVap® Centrifugal Concentrator and analyzed by RP-HPLC as described above.

### Enzyme-linked immunosorbent assay (ELISA)

Microplates (96 wells) were coated with 100 μl *H. curtus* venom proteins (2 μg/ml in 0.1 M Na_2_CO_3_-NaHCO_3_, pH 9.6) per well overnight at 4 °C. The unbound proteins were washed off with PBST (0.05% Tween-20 in 10 mM PBS, pH 7.4) and the plate was blocked with 150 μl 2% BSA in PBST at 37 °C for 1 h. After washing three times, 100 μl of serially diluted horse serum/commercial *B. multicinctus*/*N. atra* antivenom (initial concentration 4 μg/μl) in PBST containing 1% BSA was added into each well and incubated at 37 °C for 1 h. Subsequently, the plate was washed again and 1:10000 diluted HRP-labelled anti-horse IgG (Sigma) was added into each well and incubated at 37 °C for 1 h. Finally, the unbound secondary antibodies were thoroughly rinsed from the plate with PBST. Aliquots (100 μl) of substrate solution (0.5 mg/ml OPD and 0.006% H_2_O_2_ in 0.15 M citrate buffer, pH 5.0) was added to each well and incubated at room temperature for 20 min. The reaction was stopped with 50 μl 2.5 M sulphuric acid and the absorbance was recorded at 490 nm using a SpectraMax 384 microplate reader (Molecular Devices).

### Western blotting

Venom proteins of *H. curtus* were separated by 18% SDS-PAGE under reduced conditions according to Laemmli (1970). After electrophoresis, the proteins on the gel were either transferred to a PVDF membrane (0.45 μm, Millipore) in a semi-dry system (Bio-Rad) or stained with CBB R-250. The membrane was then blocked with 5% BSA in TBST (20 mM Tris-HCl pH 8.0, containing 150 mM NaCl and 0.05% Tween-20) overnight at 4 °C. After washing with TBST, the membrane was incubated with 1:500 diluted commercial antivenom at 37 °C for 1 h. Then, the membrane was washed and incubated with 1:3000 diluted HRP-labelled antihorse IgG at 37 °C for 1 h. After washing off the unbound secondary antibodies, the membrane was incubated with Pierce™ ECL western blotting substrate and exposed to X-ray film. The film was scanned using a UMax2100 densitometer (Novax Technologies) and analyzed with Tan4100 software. In addition, the protein bands in the CBB R-250 stained gel were excised and identified by LC-MS/MS according to the procedure above mentioned.

### Statistical analyses

To detect the correlation between mRNA and protein level abundance of individual genes for each toxin family, the data were centred log-ratio (clr) transformed prior to analyses according to Rokyta et al. [73]. Two coefficients (Spearman’s rank correlation coefficient and Pearson’s correlation coefficient) used for assessment of correlation were calculated by non-parametric correlation and linear regression analyses using Statistica 8.0. The significance level was set at α = 0.05.

## Supplementary Information

**Additional file 1: Table S1.** FPKM% of protein family in the venom gland transcriptome of the spine-bellied sea snake (*H. curtus*) from the South China Sea. **Table S2.** Amino acid sequences translated from toxin unigenes in the venom gland transcriptome of the spine-bellied sea snake (*H. curtus*) from the South China Sea. Sequences with partial CDS are indicated in italic. **Table S3.** Sequences used for positive selection detecting. **Table S4.** Identification of protein bands from whole venom separated by SDS-PAGE.

**Additional file 2: Figure S1.** Original images of the SDS-PAGE profiles of venom fractions separated by RP-HPLC (1–11 peak number). **Figure S2.** Original images of SDS-PAGE profiles of whole venom protein (left panel) and cross-reaction between *H. curtus* venom and commercial antivenoms by western blotting (right panel) (Hcu: *H. curtus*).

## Data Availability

The venom-gland transcriptome raw data were submitted to the NCBI Sequence Read Archive (SRA) under the accession number PRJNA670846.

## Abbreviations

3-FTx: Three finger toxin
5′NT: 5′ nucleotidase
AchE: Acetylcholinesterase
AP: Aminopeptidase
Bm: *B. multicinctus*
CREGF: Cysteine-rich EGF-like domain
CRISP: Cysteine-rich secretory protein
CTL: C-type lectin
HA: Hyaluronidase
*dN*: Nonsynoymous substitution rate
*dS*: Synonymous substitution rate
LAAO: l-amino acid oxidase
LNX: Long chain α-neurotoxin
Na: *N. atra*
NGF: Nerve growth factor
PAML: Phylogenetic analysis by maximum likelihood
PDE: Phosphodiesterase
PLA_2_: Phospholipase A_2_
PLB: Phospholipase B
QC: Glutaminyl-peptide cyclotransferases
SNX: Short chain α-neurotoxin
SVMP: Snake venom metalloproteinase
SVSP: Snake venom serine proteinase
VEGF: Vascular endothelial growth factor
VF: Venom factor
