# Supplementary material for: Venom-gland transcriptomic, venomic, and antivenomic profiles of the spine-bellied sea snake (Hydrophis curtus) from the South China Sea
Source: BMC Genomics. 2021 Jul 8;22:520. doi: 10.1186/s12864-021-07824-7 (PMC8268360; doi:10.1186/s12864-021-07824-7)

**Figure S1.** Original images of the SDS-PAGE profiles of venom fractions separated by RP-HPLC (1-11 peak number).


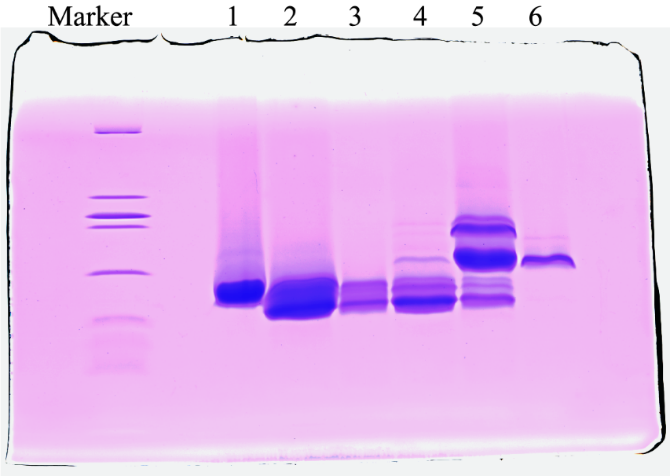

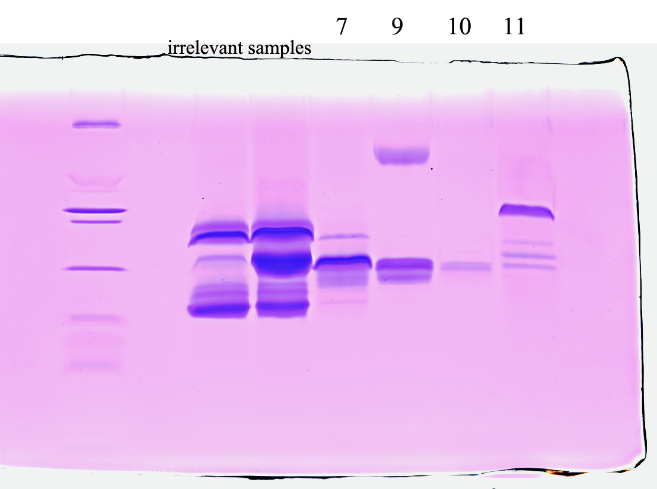

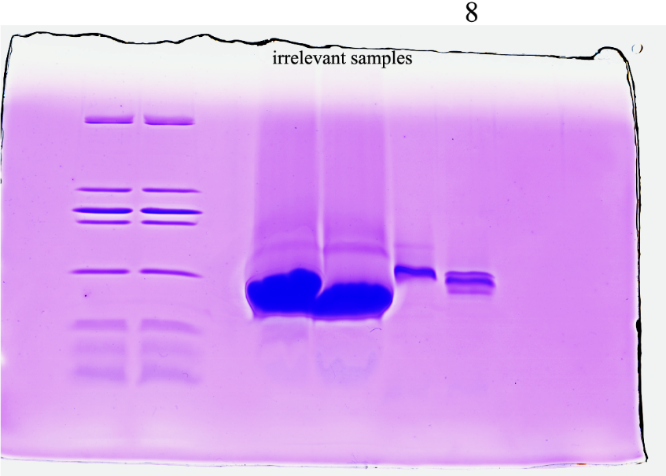


**Figure S2.** Original images of SDS-PAGE profiles of whole venom protein (left panel) and cross-reaction between *H. curtus* venom and commercial antivenoms by western blotting (right panel) (Hcu: *H. curtus*).


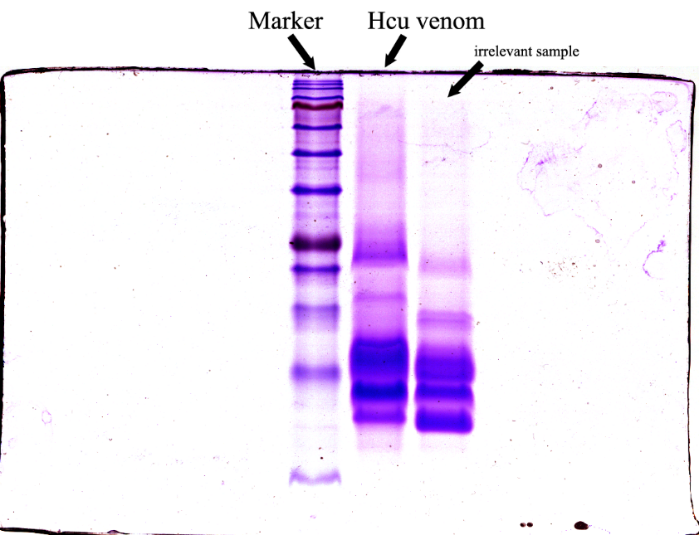

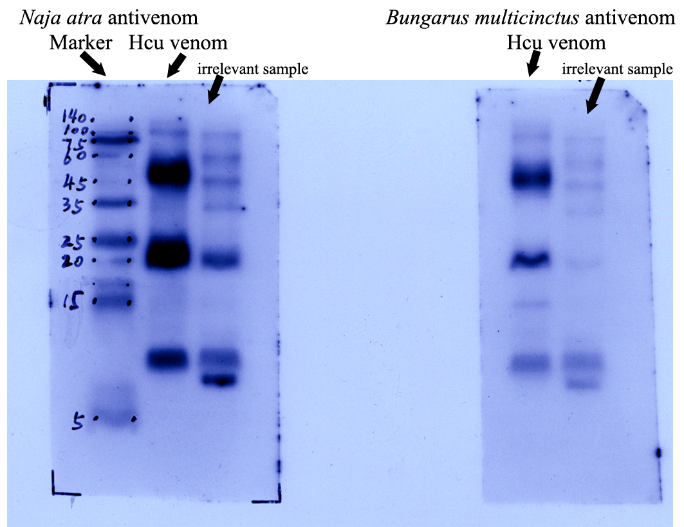

Supplement: Supplementary file 2 — Additional file 2: Figure S1. Original images of the SDS-PAGE profiles of venom fractions separated by RP-HPLC (1–11 peak number). Figure S2. Original images of SDS-PAGE profiles of whole venom protein (left panel) and cross-reaction between H. curtus venom and commercial antivenoms by western blotting (right panel) (Hcu: H. curtus). [file 12864_2021_7824_MOESM2_ESM.docx]
